# Neurogenic Effects of Inorganic Arsenic and Cdk5 Knockdown in Zebrafish Embryos: A Perspective on Modeling Autism

**DOI:** 10.3390/ijms25063459

**Published:** 2024-03-19

**Authors:** Qiang Gu, Jyotshna Kanungo

**Affiliations:** Division of Neurotoxicology, National Center for Toxicological Research, U.S. Food and Drug Administration, 3900 NCTR Road, Jefferson, AR 72079, USA

**Keywords:** arsenic, zebrafish, Sonic hedgehog, autism, motor neuron

## Abstract

The exact mechanisms of the development of autism, a multifactorial neurological disorder, are not clear. The pathophysiology of autism is complex, and investigations at the cellular and molecular levels are ongoing to provide clarity. Mutations in specific genes have been identified as risk factors for autism. The role of heavy metals in the pathogenesis of autism is subject to many studies and remains debatable. Although no exact neuronal phenotypes have been identified linked to autistic symptoms, overproduction and reduction of specific neurons have been implicated. A growing literature on generating genetic and non-genetic models of autism aims to help with understanding mechanistic studies that can explain the complexity of the disorder. Both genetic and non-genetic methods of zebrafish have been used to model autism. For several human autism risk genes, validated zebrafish mutant models have been generated. There is growing evidence indicating a potential link between autism and inorganic arsenic exposure. We have previously shown that inorganic arsenic induces supernumerary spinal motor neurons via Sonic hedgehog (Shh) signaling pathway, and Cdk5 knockdown causes an overproduction of cranial and spinal motor neurons in zebrafish. Here, in this review, we provide a perspective on what these findings of neurogenic phenotypes mean in terms of dysregulated pathways of motor neuron development and their applicability to understanding cellular and molecular underpinnings of autism.

## 1. Introduction

Autism spectrum disorder (ASD), commonly known as autism, is a complex neurodevelopmental disorder [1,2]. Due to the broad spectrum of this neurodevelopmental disorder, a diagnosis of autism remains a challenge and is based on the individual’s behavioral patterns and developmental history, while the severity and variability of the symptoms can vary among individuals [3,4]. Autism causes motor defects such as difficulty in walking, postural irregularities causing clumsiness, and balance issues, while non-motor defects include memory and cognitive deficits, irritation, anxiety, and aggressive behavior [5].

Over the past two decades, the prevalence of autism reported worldwide has been steadily increasing. In 2000, according to Autism and Developmental Disabilities Monitoring (ADDM), the incidence of autism was estimated to be 1 in 150 children. In 2006, the incidence was 1 in 110 children, and by 2008, the incidence had increased to 1 in 88 children [6]. A recent estimate shows that more than 70 million people, i.e., 1.5% to 2% worldwide, suffer from autism [7]. In 2023, the Center for Disease Control (CDC) reported that the incidence increased to 1 in 36 (2.8%) [8].

With hitherto unknown specific causes, autism, a multifactorial neurodevelopmental disorder, is found to be highly heritable, and many studies reveal that genetic factors (the involvement of many genes) as well as environmental factors are the major contributors/risk factors for the development of autism [2,9,10]. Environmental chemicals can contribute to human diseases, including autism [11]. Mounting evidence indicates that autism results from complex interactions between genes and the environment [12,13]. A systematic review and meta-analyses of 53 studies involving 5054 children described an association between autism and heavy metal exposure including arsenic, cadmium, mercury, and lead [14]. Arsenic is considered a potential contributor to the development of autism [15]. Multiple studies have shown an association of arsenic with autism (reviewed in [14]). An epidemiological study on 397 autism cases and 1034 controls under the Norwegian Mother, Father and Child Cohort Study showed a positive association between prenatal exposure to arsenic and autism risk [16] A report showed that populations living closer to industrial facilities that emit heavy metals such as arsenic, lead, and mercury to the air had a higher occurrence of autism [17]. Epidemiological studies show that arsenic exposure during critical periods of neurodevelopment could pose as an environmental risk factor for autism development [18,19]. Significantly higher levels of arsenic in the urine [20,21,22,23], blood [24], and hair [22] of children with autism have also been reported [20,21]. Arsenic exposure through drinking water at 10–50 ppb has been shown to cause peripheral neuropathy in humans [25]. In children, central nervous system (CNS) impairment may occur at ≥ 50 ppb [26]. Mice, after prenatal exposure to arsenic, showed an increased number of pyramidal neurons of the prelimbic cortex, which has been linked to behavioral inflexibility in adulthood due to cortical disarrangement [27]. Although arsenic’s effects on specific neurons in the brain have been well studied, very few studies have focused on its effects on motor neurons. An epidemiological study in arsenic-contaminated regions showed a 16.7% higher risk of mortality associated with motor neuron disease [28]. Additional epidemiological studies show a potential association of heavy metals, including arsenic, with autism (reviewed in [14]) that warrants further studies in order to determine whether there is a direct link between these heavy metals and autism.

Mutations in cyclin-dependent kinase 5 (Cdk5) have been reported in patients with non-syndromic intellectual disability [29]. Selective loss of Cdk5 in the dorsolateral striatum of mice caused increased locomotor activity with attenuated motor learning [30]. Valproic acid, prenatal exposure to which causes autism-like behavioral abnormalities and brain malformation in animal models including zebrafish [31,32], downregulates Cdk5 activity in cultured mouse neurons [33]. The effects of the downregulation of Cdk5 activity on specific neuron development (a specific neuronal phenotype) can help unravel cellular and molecular mechanisms behind autism-like symptoms in animal models with follow-up studies.

One of the pathological mechanisms of autism underlies impaired functions of specific brain regions and dysfunctional neural circuits [13]. For example, functional studies of an autism-associated gene, *Shank3*, a synaptic scaffold protein that is enriched at the postsynaptic excitatory synapses [34], show that mice lacking *Shank3* not only exhibit hypertrophy of the striatum but also experience decreased cortico-striatal excitatory synaptic transmission and show repetitive behaviors [35]. Lately, various animal modeling studies have revealed several types of viable mutations, which can shed light on the underlying mechanisms of autism pathogenesis [13]. Due to the evolutionary conservation of the developmental processes of the nervous system between zebrafish and mammals, zebrafish are used to investigate autism using both genetic and non-genetic methods (reviewed in [2]). Zebrafish exhibit similar behavioral responses as in mammals, such as social interactions and preference, as well as repetitive behaviors, making it possible to model phenotypes with ASD-like symptoms [36]. A list of zebrafish mutant lines for twelve autism risk genes has been curated (https://www.sfari.org/resource/zebrafish-models/ (accessed on 10 December 2023)) by the Simons Foundation for Autism Research Initiative (SFARI). As an alternative animal model, data from zebrafish autism studies can add to the knowledge gap that exists in mammalian studies, reveal mechanistic pathways, and help with drug discovery. 

Chronic arsenic exposure altered social behavior, a characteristic of autism, in juvenile zebrafish, which was ameliorated by the antioxidant N-acetylcysteine [37]. In zebrafish larvae, arsenic caused motor behavioral deficit as well as mild impairment in behavior towards color preference [38]. Transgenerational changes in motor activity and anxiety-like behavior upon arsenic exposure, accompanied by a reduction in brain-derived neurotrophic factor level and increased histone methylation, have been reported in zebrafish [39]. Arsenic caused hypoactivity of zebrafish larvae in a photomotor response assay [40]. Anxiety-like behavior and alteration in long-term memory have also been reported in adult zebrafish upon arsenic exposure [41]. Based on existing studies both in mammals and zebrafish, this review provides a perspective on what the neurogenic phenotypes indicate in terms of modeling autism.

## 2. Risk Genes of Autism and Zebrafish

Many reliable risk genes for autism development have been discovered (reviewed in [2]), and approximately 5% of autism cases result from single-nucleotide polymorphisms (SNPs) in genes such as *NLGN3*, *NLGN4*, *NRXN1*, *MECP2*, *SHANK3*, *FMR1*, *TSC1/2*, and *UBE3A* (reviewed in [2]). Genetic alterations that can increase the risk of autism include changes in UBE3A, a ubiquitin protein ligase E3A [42], MAPK3 (mitogen-activated protein kinase 3) [43], as well as an increase in the copy number variants, such as single nucleotide polymorphisms (SNPs), for example, in the chromosomal region 15q11-q13.3 [42]. In addition to this, epigenetic mechanisms that include histone modification, DNA methylation, chromatin remodeling, and micro-RNA activity are involved in the regulation of social behavior in autism [44].

A study on zebrafish using high-throughput functional analysis of 10 autism risk genes identified convergence of dopaminergic and neuroimmune pathways [45]. The functions of 12 autism genes (*ARID1B*, *CHD8*, *CNTNAP2*, *DYRK1A*, *GRIN2B*, *FMR1*, *MECP2*, *NRXN1*, *PTEN*, *SCN2A*, *SHANK3*, and *SYNGAP1*) have been studied in zebrafish [2]. In zebrafish embryos, morpholino (MO)-mediated knockdown of CHD8, a chromatin-binding protein that targets many other autism-related genes, results in macrocephaly consistent with human autism cases with CHD8 loss of function [46]. Knockdown of FMR1 in zebrafish larvae resulted in autism-like behavior [47] similar to the valproic-acid treated zebrafish larvae [48]. *MECP2* knockout in zebrafish caused behavioral and motor deficits [49], and MECP2 knockdown suppressed neural precursor cell differentiation [50]. Double mutation in *CNTNAP2a/b* zebrafish caused reduced GABAergic neurons [51]. Zebrafish *DYRK1A* mutants have microcephaly [52]. Homozygous recessive loss-of-function mutation in *scn1alab*, a voltage-gated sodium ion channel, caused abnormal neuronal firing, hyperactivity, and convulsive behaviors in zebrafish that are consistent with effects shown in mice and humans [53]. *Shank3a/b* knockout zebrafish embryos/larvae as well as adults had reduced revels of synaptic proteins and displayed robust autism-like behaviors with reduced locomotor activity [54]. *Syngap1a/b* knockdown embryos had significantly decreased GABAergic neurons [55]. These studies emphasize the utilization of the zebrafish model for autism studies that can reveal useful information on this complex neurodevelopmental disorder.

## 3. Autism and Overproduction and Reduction of Specific Neurons

A preliminary study reported brain overgrowth and an excess number of neurons in the pre-frontal cortex of autistic male children [56]. An overproduction of upper-layer neurons in the neocortex in mice has been shown to lead to autism-like features, suggesting a causal link between the overproduction of certain neurons and autism, which offers some insight into the etiology of the disorder [57]. It has been reported that although the number of mature neurons of the human amygdala increases from childhood into adulthood under normal development, in autistic individuals, an initial excess of neurons in the amygdala during childhood is followed by a reduction of neurons in adulthood [58]. Such developmental anomalies might offer critical information on the etiology of autism. Decreased cortical interneurons in autism have also been reported, indicating that interneuron hypofunction could be a primary driver of erroneous circuit engagement and dysfunction in autism [59]. Furthermore, a study on organoids derived from induced pluripotent stem cells from patients with Fragile X Syndrome (a known cause of autism) showed a lower density of GABA-expressing neurons [60].

Autopsies of patients with autism have shown significant structural changes of their brains, e.g., altered grey and white matter ratios, increased neuronal numbers accompanied by reduced neuronal body volume, increased numbers of glia, and changes in dendritic spines and cerebral vasculature [61]. Longitudinal imaging studies on toddlers (18 and 60 months old) with autism revealed an enlarged amygdala [62], and children with autism had 67% more neurons in the prefrontal cortex [56]. On the other hand, Purkinje cells were decreased in the cerebellar hemispheres of autistic individuals, who also had reduced numerical density of neurons in the putamen and nucleus accumbens [63]. Brain tissues of individuals with autism have supernumerary neurons in the cerebral cortical subplate [64]. The increased brain size in subjects with autism [65] has been attributed to an increased number of neurons or increased neuropil when there was no change in neuron numbers [66]. Malformations of the CNS resulting from such abnormal neurodevelopment (lack of or over-abundance of specific neurons) can lead to autism, cognitive delay, and intractable epilepsy [67,68].

Moreover, early assessments of autism show striatal hypertrophy with reduced amygdala volume albeit increased neuronal density in the region covering the medial, central, and lateral nuclei that plays critical roles in anxiety, fear conditioning, and social behavior [13,69,70]. Additionally, prenatal exposure to valproic acid, which, clinical evidence indicate, has a strong association with autism [71,72], enhanced untimely embryonic neurogenesis in mice, leading to a depletion of the neural precursors and resulting in decreased levels of adult hippocampal neurogenesis [73]. In zebrafish embryos, valproic acid adversely affected neurogenesis in the optic tectum [74], reduced midbrain size, and reduced the number of neuronal progenitors, along with perturbations in the secondary motor neuron neurite development [75]. Modeling the genetic as well as environmental aspects in zebrafish embryos can offer an ideal system for an in-depth investigation of the potential mechanisms of autism development, since manipulation of individual risk genes in these embryos may lead to the identification of phenotype-based mechanistic pathways.

## 4. Arsenic and Zebrafish Motor Neurons: Relevance to Autism

**Figure 1:**
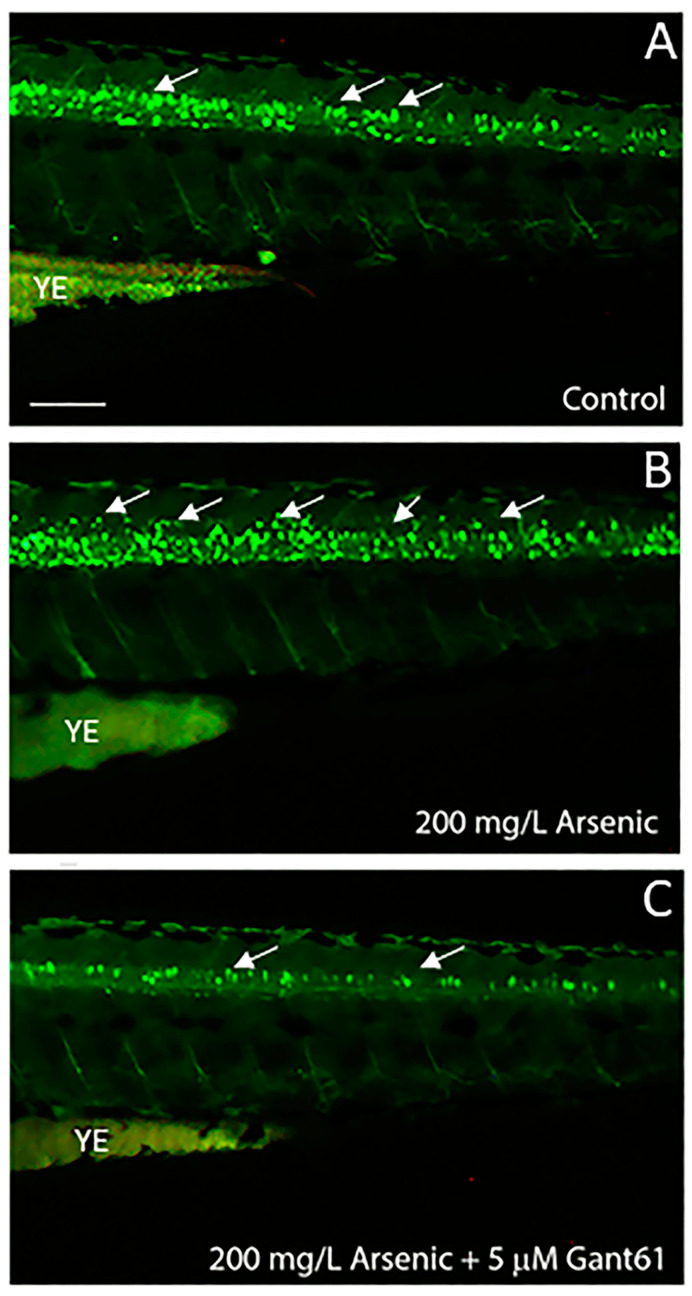
Inorganic arsenic-induced supernumerary motor neuron development is inhibited by the Shh inhibitor, Gant61. Five hours post fertilization (hpf), transgenic embryos (*hb9-GFP*) that express green fluorescent protein (GFP) in the motor neurons were exposed to 200 mg/L of sodium arsenite (internal concentration of 387.8 ± 26.9 pg/embryo). Fluorescent images of spinal cord regions of the 72 hpf embryos are shown for control (**A**), 200 mg/L sodium arsenite-treated (**B**), 200 mg/L sodium arsenite with 5 μM Gant61-treated (**C**). Arrows indicate GFP-expressing motor neurons. YE indicates yolk extension (Adapted from Kanungo et al. [76]).

Recently, we reported that arsenic induced supernumerary spinal motor neurons in transgenic (*hb9-GFP*) zebrafish that express green fluorescent protein (GFP) in the motor neurons via Sonic hedgehog pathway (Figure 1A–C) and also increased the density of tyrosine hydroxylase-positive dopaminergic neurons [76]. However, arsenic did not alter the density of serotonergic neurons [76]. In vertebrates, the formation of motor neurons depends on Hedgehog (Hh) signaling, which is mediated by Gli zinc finger proteins [77].

There are three Hh family members, Sonic Hedgehog (Shh), Indian Hedgehog (Ihh) and Desert Hedgehog (Dhh). These three proteins activate a common signaling pathway, called Hh signaling, and arsenic activates Hh signaling [78]. Shh, a secretory protein acts as a developmental morphogen, and Shh signaling plays an integral role in embryogenesis including neurodevelopment and neurodegeneration [79]. The Shh signaling pathway plays an important role in development [80]. Vertebrate Patched (a receptor) binds to the Shh ligand [81]. Such binding relieves the inhibitory effect of Patched on a seven-transmembrane protein, Smoothened, resulting in transcription of the Gli transcription factors, including Gli2 [82,83]. Gli2, a positive regulator of Shh signaling, is then activated [84,85,86]. However, supernumerary motor neuron development is inhibited by the Shh signaling inhibitor Gant61 [76]. While Gli1 can induce Nkx2.1-positive ventral forebrain neuron development, both Gli1 and Gli2 can induce Hb9-positive spinal motor neuron development [87]. In arsenic-treated zebrafish, Patched gene expression was not altered [88]. Whether protein levels of Patched and Shh changed in arsenic-treated embryos remains under investigation.

**Figure 2:**
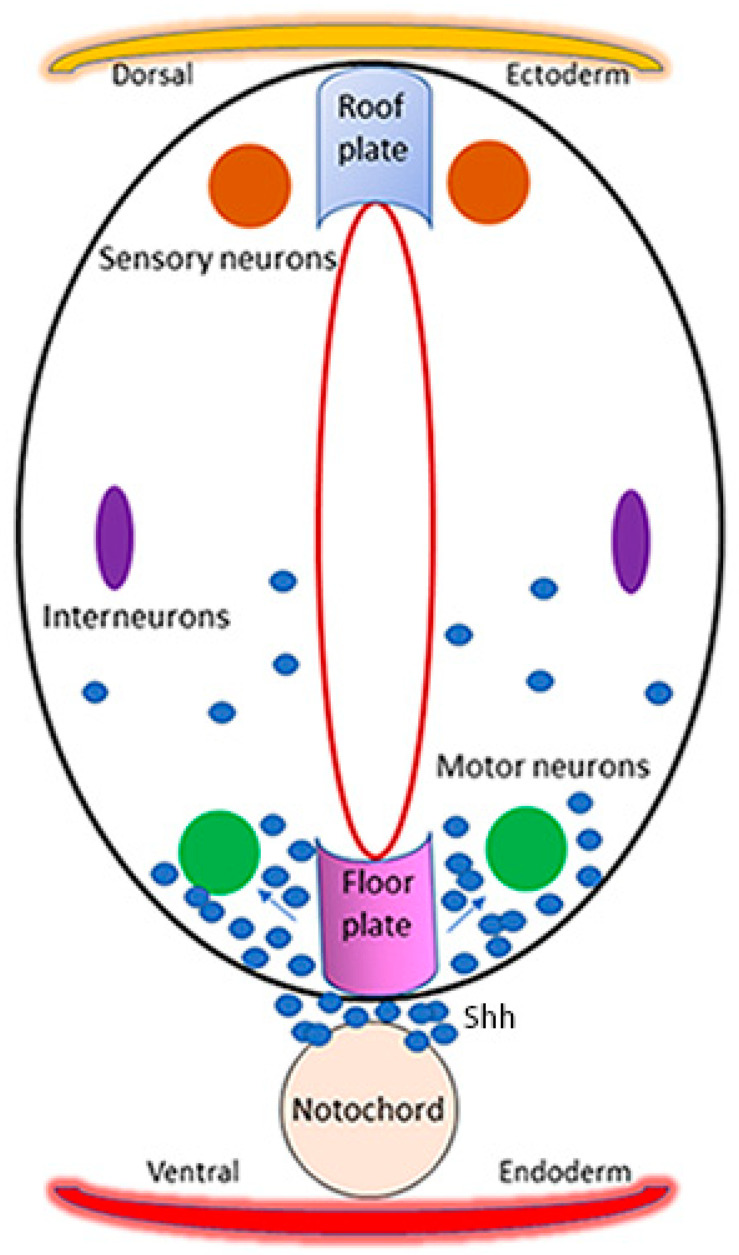
Schematic presentation of motor neuron development in zebrafish. The neural tube develops from the neural plate with the dorsal location of the sensory neurons, intermediate location of the interneurons, and ventral location of the motor neurons. The dorsal ectoderm above the roof plate and the ventral endoderm below the notochord are shown. Sonic hedgehog (Shh) is expressed in the floor plate and notochord, which triggers the development of motor neurons from the neuronal progenitors of the floor plate.

Shh is secreted from the notochord and is critical for the development of the motor neurons in vertebrates [87] (Figure 2). Shh signaling activates Gli genes, which are known to affect motor neuron development and positioning in the spinal cord during early development of vertebrates [89,90]. In the CNS, Shh plays a critical role in ventral specification along the neural axis. Overexpression of Shh in the spinal cord has been shown to alter the positioning of the motor neurons and results in the aberrant structure of the motor column [90]. Misexpression of Shh can induce the differentiation of floor plate cells including motor neuron differentiation at ectopic locations in the spinal cord in vertebrate embryos [91,92,93]. A schematic presentation of motor neuron development in zebrafish pertaining to the Shh signaling pathway is shown in Figure 2. The cross-sectional view of the neural tube flanked dorsally by the ectoderm and ventrally by the endoderm shows the location of the roof plate, sensory neurons, interneurons, motor neurons, floor plate, and notochord, the latter producing the Shh that induces motor neuron development (Figure 2). This pathway of motor neuron development is conserved in vertebrates [94].

Based on rodent studies, it has been postulated that the motor dysfunction caused by arsenic ingestion may be a consequence of arsenic’s direct influence on motor neurons rather than other processes, such as demyelination [95]. In children with autism, significantly higher levels of serum Shh protein have been reported [96]. A low dose of arsenic can induce Hh signaling in vitro and in vivo [78]. Dysregulation of Shh signaling leads to many physiological changes that precede neurological disorders such as autism and cognitive decline (reviewed in [97]). These data suggest a mechanistic link between arsenic, motor dysfunction, and some of the symptoms commonly observed in ASD. In mouse models of autism, mechanisms involving cellular and synaptic functions of the neurons of the peripheral somatosensory system, as well as spinal cord neurons, have been shown to contribute to tactile over-reactivity [98,99,100,101,102]. Further studies on arsenic-induced supernumerary neurons in zebrafish are needed to explore the molecular and behavioral changes related to autism. The questions that remain to be answered are whether arsenic alters Shh expression at the gene and/or protein level and whether downstream Gli genes are induced by arsenic.

In the *hb9:GFP* zebrafish embryos, it was difficult to discern brain motor neurons as opposed to those in the spinal cord [76]. Future research using high-resolution microscopy might reveal a clearer picture.

## 5. Cdk5 Knockdown and Zebrafish Motor Neurons: Relevance to Autism

**Figure 3:**
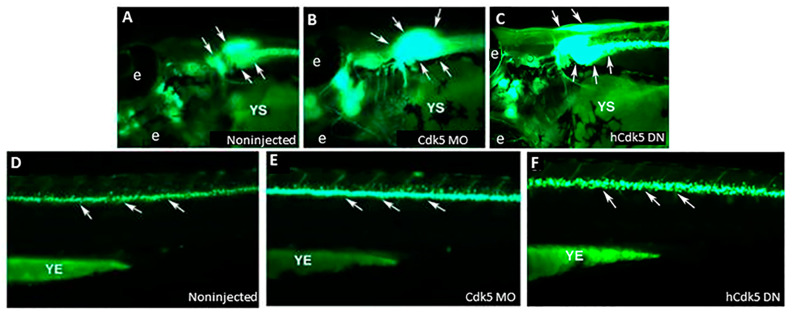
Cdk5 knockdown through microinjection of Cdk5 morpholino and dominant negative human Cdk5 (hCdk5 DN) mRNA caused supernumerary motor neuron generation in zebrafish embryos. Images of live 72 hpf *islet-1*–GFP transgenic zebrafish embryos show motor neurons in the brain; (**A**) noninjected (vehicle only) control, (**B**) Cdk5 morpholino (MO)-injected, and hCdk5 DN mRNA-injected (**C**); spinal regions of (**D**) noninjected (vehicle only) control; (**E**) Cdk5 morpholino (MO)-injected embryos; and (**F**) hCdk5 DN mRNA-injected embryos. Arrows indicate the GFP-expressing motor neuron populations in the brain (upper panel) and spinal cord (lower panel). YS indicates the yolk sac; e indicates the eye; YE indicates yolk extension. (Adapted from Kanungo et al. [108]).

Cdk5 is a member of the family of serine/threonine cyclin-dependent kinase, which is highly expressed in neurons [103]. A multi-functional protein kinase, Cdk5 regulates a wide range of neuronal functions, including neuronal survival and migration and plays a critical role in neuronal differentiation [103]. Additionally, Cdk5 is essential in regulating a number of cellular processes of the nervous system including protein trafficking, neurite and synapse development, dopaminergic function, learning, and memory [104,105]. Dysregulation of Cdk5 activity can cause a wide range of pathological processes affecting the nervous system development, leading to neurodegeneration [103]. Suppressing Cdk5 activity in cultured cortical neurons leads to compromised neurite outgrowth, whereas ectopic expression of exogenous Cdk5 and its regulator p35 produce longer neurites [106]. Cdk5-null mice that are embryonically lethal show an aberrant development of the cortex and cerebellum [107]. We have previously shown that the suppression of Cdk5 activity through MO-mediated Cdk5 knockdown or overexpression of the dominant negative human Cdk5 (hCdk5 DN) mRNA generated supernumerary motor neurons in vivo in zebrafish [108] (Figure 3A–F). In the *islet-1-GFP* transgenic zebrafish embryos that express GFP in the motor neurons, morpholino-mediated Cdk5 knockdown (translational inhibition) and hCdk5 DN-mediated suppression of Cdk5 activity caused supernumerary motor neuron generation in both cranial and spinal regions (Figure 3) [108]. In these embryos, Cdk5 activity was significantly reduced [108]. Although using a single morpholino may not be sufficient to provide a conclusive statement about a gene function in studies conducted lately, as the specificity of the morpholinos needs more controls, overexpression of the kinase-dead hCdk5 DN mRNA increasing motor neuron density further strengthens the finding (Figure 3). In agreement with this, the study also showed that overexpression of Cdk5 mRNA reduced the motor neuron density in the zebrafish embryos compared to the control [108].

Cdk5 has been implicated in the pathogenesis of various neurological disorders including autism [109]. Downregulation of Cdk5 has been associated with attention deficit and hyperactivity disorder [110], epilepsy [111], and schizophrenia [112]. Cdk5 rescued hippocampal synaptic plasticity in a mouse model of Fragile X Syndrome, a genetic form of intellectual disability associated with epilepsy, autism, and mood disorders, suggesting that activation of Cdk5 activity might be a pharmacological tool to treat Fragile X Syndrome [113]. However, no association between polymorphisms in Cdk5 with autism was found in a Chinese Han population [114]. On the other hand, studies explored a severe neurodevelopmental disorder that was characterized by intellectual disability, early-onset seizures, and autistic features resulting from mutations in the X-linked cyclin-dependent kinase-like 5 (CDKL5) gene [115,116,117]. The mechanism behind this link between the CDKL5 mutation and autistic behavior is not known.

We have shown that Cdk5 activity is significantly reduced in zebrafish *mindbomb 1* (*Mindbomb E3 ubiquitin protein ligase 1*) mutants [118]. The *mindbomb 1* (*Mib1*) gene was first identified as an E3 ubiquitin ligase in zebrafish through genetic mutagenesis screens [119]. In zebrafish, *Mib1* positively regulates the Notch pathway [119] necessary for cell fate specification [120]. While *Mib1*-null mice are embryonically lethal [121], the loss-of-function zebrafish mutant (*mindbomb 1*) exhibits developmental defects due to a loss of Notch signaling-induced lateral inhibition, thus resulting in a neurogenic phenotype characterized by increased supernumerary primary neurons [119]. In addition to defects in neurogenesis, severe defects in angiogenesis and somitogenesis occur in zebrafish *Mib1* mutants [122,123,124]. In humans, *Mib1* mutations contribute to congenital heart disease through disruptions in heart development [125]. *Mib1* homozygous mutant zebrafish do not live beyond four days, whereas *Mib1* heterozygotes (a recessive mutation) are able to survive and breed [126].

*Mindbomb* mutant zebrafish exhibit spontaneous seizures accompanied by altered gene expression in the GABA signaling pathways [127]. Loss of function due to point mutations in human ubiquitin E3A ligase has been reported in patients with autism [128]. Additionally, small deletions or mutations in the human ubiquitin E3A ligase gene have been linked to autism [129]. *Mib1* has been shown to regulate neurite morphogenesis by interacting with Cdk5 and its regulator p35 [130]; however, it is not clear why *mindbomb* mutant zebrafish have reduced Cdk5 activity [118]. A potential pathway of the reduction in Cdk5 activity in the *mindbomb* mutant zebrafish has been proposed, which suggests that overexpression of Cdk5 beyond a threshold limit can reduce its own activity [118]. This study indicated that reduction of Cdk5 activity but not Cdk5 mRNA level itself is critical for the overproduction of primary neurons, and Notch inhibition (*mindbomb/Mib1* mutant) is upstream of the downregulation of Cdk5 activity [118].

Mib1 ubiquitinates and induces the degradation of survival of motor neuron proteins (SMNs), and *Mib1* knockdown increases SMN protein levels in HEK-293T cells, suggesting a beneficial effect on the survival of motor neurons [131]. Similar to *Mib1* mutation, arsenic has been shown to block Notch signaling in a human small-cell lung cancer cell line [132,133]. Whether arsenic inhibits Notch signaling in zebrafish, which could be responsible for the neurogenic phenotype we have reported [76], warrants further studies that would reveal divergent or convergent pathways linking the phenotypes to the upstream events.

## 6. Conclusions

**Figure 4:**
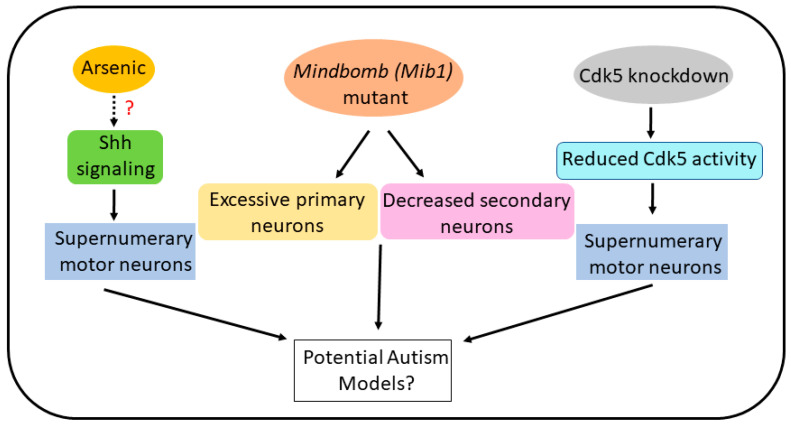
Schematic presentation of scenarios occurring in zebrafish treated with arsenic, the *mindbomb (Mib1)* mutant, and morpholino (MO)-mediated Cdk5 knockdown or human dominant negative Cdk5 mRNA expression that caused decreased Cdk5 activity. How arsenic could modulate Shh signaling is not known. Nonetheless, supernumerary (excessive) neurons (primary and motor neurons) resulting from these cases may be used to model and study cellular and molecular mechanisms of autism in a lower vertebrate like zebrafish.

An overproduction and reduction of specific neurons have been reported in autism, which can potentially explain the excitation/inhibition imbalance displayed in individuals with this serious and complex disorder [59]. In the phenotypes discussed above, data are lacking on whether the overproduction of tyrosine hydroxylase positive neurons in the brain by arsenic or motor neurons in the spinal cord by Cdk5 knockdown and arsenic in zebrafish occurred while there was reduction in other types of neurons (e.g., interneurons). Arsenic and Cdk5 knockdown, inducing supernumerary motor neurons and producing a similar outcome in motor neuron development, indicates that such phenotypes can occur through many different mechanisms and may cause an imbalance in specific neuron functions, e.g., excitatory to inhibitory imbalance—a hallmark of autism (Figure 4). Arsenic also increased tyrosine hydroxylase positive neurons in the brains of zebrafish embryos [76]. These phenotypes may be utilized as preclinical models for in-depth studies to demonstrate whether the alteration in the neuronal development patterns predisposes the organism to exhibit autism-like symptoms (Figure 4). Zebrafish have been utilized to model phenotypes related to autism either through genetic manipulation or chemical exposure (e.g., valproic acid) (reviewed in [2]). Although these phenotypes of zebrafish cannot completely simulate the pathological processes of autism reported in human beings, they will help to understand the triggers and molecular precursors of the development of autism. Therefore, alterations in early development of specific neurons in autism risk-gene mutants or those that are induced by chemicals need to be investigated. Furthermore, exposing specific autism risk-gene mutants of zebrafish to arsenic and examining the effects on specific neuron types would reveal deeper understanding of the multifactorial nature of the disease. While stem cell models have been able to reveal that disruptions in specific molecular processes, such as calcium and Wnt signaling, and chromatin remodeling can contribute to the pathogenesis of autism [46,134], being a vertebrate with conserved genetic and physiologic pathways [135], zebrafish carry an advantage in phenocopying cellular and behavioral aspects of autism that can reveal hitherto unknown mechanisms. The early detection of defective neuronal development would help delineate the mechanism of the role of environmental factors in autism development, which would shed light on gene/environment interactions and provide opportunities for therapeutic drug discovery. The current perspective presents a scenario that warrants further investigation of the zebrafish phenotypes with an overproduction of specific neurons in order to determine whether autism can be modeled to a certain extent, if not completely, using these embryos.

## Data Availability

No new data are reported in this review article.

## Abbreviations

ASD	Autism spectrum disorder
Cdk5	Cyclin-dependent kinase 5
CHD8	Chromodomain helicase DNA binding protein 8
FMR1	Fragile X Messenger Ribonucleoprotein 1
GFP	Green fluorescent protein
Gli	Glioma-associated oncogene, a zinc finger protein
MECP2	Methyl CpG binding protein 2
Mib1	Mindbomb E3 ubiquitin protein ligase 1
NLGN3	Neuroligin-3
NLGN4	Neuroligin-4
NRXN1	Neurexin 1
SHANK3	SH3 and multiple ankyrin repeat domains 3 (also known as proline-rich synapse-associated protein 2)
TSC1/2	Tuberous sclerosis complex 1/2
UBE3A	Ubiquitin-protein ligase E3A (also known as E6AP ubiquitin-protein ligase)
